# Nanocellulose-Based Biomaterial Ink Hydrogel for Uptake/Release of Bovine Serum Albumin

**DOI:** 10.3390/polym15040837

**Published:** 2023-02-08

**Authors:** Wan Nazihah Liyana Wan Jusoh, Denesh Mohan, Mohd Shaiful Sajab, Peer Mohamed Abdul, Hatika Kaco, Gongtao Ding, Rubiyah Baini

**Affiliations:** 1 Research Center for Sustainable Process Technology (CESPRO), Faculty of Engineering and Built Environment, Universiti Kebangsaan Malaysia, Bangi 43600, Selangor, Malaysia; 2 Department of Chemical and Process Engineering, Faculty of Engineering and Built Environment, Universiti Kebangsaan Malaysia, Bangi 43600, Selangor, Malaysia; 3 Kolej GENIUS Insan, Universiti Sains Islam Malaysia, Bandar Baru Nilai, Nilai 71800, Negeri Sembilan, Malaysia; 4 Key Laboratory of Biotechnology and Bioengineering of State Ethnic Affairs Commission, Biomedical Research Center, Northwest Minzu University, Lanzhou 730030, China; 5 Faculty of Engineering, Universiti Malaysia Sarawak (UNIMAS), Kota Samarahan 94300, Sarawak, Malaysia

**Keywords:** 3D printing, bioprinting, cellulose, liquid printing, protein

## Abstract

This study explores the potential of using nanocellulose extracted from oil palm empty fruit bunch (OPEFB) as a biomaterial ink for 3D printing. The research focuses on using nanocellulose hydrogels for the controlled uptake and release of proteins, with the specific protein solution being Bovine Serum Albumin (BSA). To provide a suitable material for the bioprinting process, the study examines the characteristics and properties of the printed hydrogels through various analyses, such as morphology, functional group, crystallinity, and compression test. Several parameters, such as initial concentration, temperature, and the presence of calcium chloride as an additional crosslinker, affect the protein uptake and release capabilities of the hydrogel. The study is important for biomedicine as it explores the behavior of protein uptake and release using nanocellulose and 3D printing and can serve as a preliminary study for using hydrogels in biological materials or living cells.

## 1. Introduction

Nanocellulose, a material derived from plant fibers, has gained increasing attention for its advanced properties and potential for commercialization in various industries, particularly in biopolymers and biomedical fields [1,2]. Malaysia, being a major producer of oil palm, has significant potential to transform underutilized biomass into valuable products [3,4]. The extraction and purification of nanocellulose from oil palm empty fruit bunch (OPEFB) using green methods is an efficient way to minimize waste and keep production costs low [5]. The high strength and mechanical properties of nanocellulose make it ideal for use in additive manufacturing (3D printing), and its potential to produce customized and detailed products [1,6].

3D printing categorizes different techniques based on the feed material and application. Most ink materials are in solid, liquid, and powder forms [7]. Previously, many inks were made up of polylactic acid (PLA), acrylonitrile butadiene styrene (ABS), polyurethane (PU), and polyethylene glycol (PEG), which experience resource depletion and are less suitable for sustainability and the environment [8,9]. Nanocellulose from OPEFB as printing ink has a higher biodegradation rate and can benefit underutilized biomass into smart and sustainable material [10,11,12]. As the demand for biopolymer printing ink increases, it is a great chance to start with nanocellulose. Biopolymer ink derived from natural resources solves some previous ink issues, having high biocompatibility for living thing applications.

Many studies have explored cellulose derivatives as biomaterial ink, such as cellulose nanocrystalline, cellulose nanofibrils, bacterial nanocellulose, and regenerated nanocellulose [13,14,15,16,17]. However, the use of nanocellulose derivatives as the biomaterial ink for the uptake and release of Bovine serum Albumin (BSA) protein solution with 3D printing has not been extensively discovered yet. The cellulose nanofibrils (CNF) biomaterial ink derived from OPEFB is used for the uptake and release of BSA together with 3D printing. The capability for protein uptake and release will directly explain the potential for other biological applications involving living cells in the near future. Furthermore, CNF properties show good characteristics for the shape fidelity of the 3D-printed hydrogel with customized design. The structure of hydrogel, which is almost similar to the extracellular matrix, is favorable for the biomaterial entrapment and growth of living cells [16,18].

## 2. Materials and Methods

### 2.1. Materials

Oil palm empty fruit bunch fiber (OPEFB) with a size range from 106 to 500 μm is the raw material used to extract cellulose. For the cellulose preparation and cellulose ink, 95% formic acid, 30% hydrogen peroxide (H_2_O_2_), iron (II) prepared from iron (II), sulfate heptahydrate (Fe_2_SO_4_·7H_2_O), lithium hydroxide, urea, sodium carboxymethyl cellulose, sulphuric acid (H_2_SO_4_) and calcium chloride (CaCl_2_) were obtained from Merck, Darmstadt, Germany. The adsorption and release of protein were conducted by using bovine serum albumin (BSA, lyophilized powder, ≥90%, agarose) and phosphate buffer saline (PBS) obtained from Sigma Aldrich, Boston, MA, USA.

### 2.2. Extraction of Cellulose

The extraction of cellulose from oil palm empty fruit bunch (OPEFB) fibers was conducted in accordance with previous studies [19,20]. The process began with removing lignin from OPEFB using an organosolv extraction method. The experiment used a ratio of 30:1 formic acid to OPEFB fibers in a three-necked, flat-bottomed flask equipped with a condenser to control heat transfer. The flask was placed on a digital hotplate magnetic stirrer (IKA RCT Basic, IKA Staufen, Germany) at a constant temperature of 90 °C for 2 h and at a stirring rate of 800 rpm. The OPEFB pulp and supernatant from the organosolv extraction were separated using a vacuum filter (IKA MVP10 Basic, IKA, Staufen, Germany) and washed several times with deionized water.

After the lignin extraction, the OPEFB pulp was further purified using catalytic oxidation to remove the remaining hemicellulose and unextracted lignin. This process used a low concentration of hydrogen peroxide (4%) in the presence of Fe(II) ions from iron(II) sulfate heptahydrate, Fe_2_SO_4_·7H_2_O. The catalytic oxidation was carried out at 90 °C for 24 h, with a small amount of hydrochloric acid added to stabilize the solution. The mixture was then separated using a vacuum filter and washed repeatedly with deionized water. The purified cellulose pulp was stored in a chiller at 4 °C, and its purity and lignin content were monitored using National Renewable Energy Laboratory (NREL) standards.

### 2.3. Preparation of Cellulose Nanofibers and CNF and CMC

The cellulose isolated in the previous step was mixed with deionized water (0.7 wt%) and fibrillated using a high-speed homogenizer (IKA T25 Digital, Staufen, IKA Germany) at 37,000 rpm for 30 min in a controlled cycle to produce cellulose nanofibrils (CNF). The temperature of the solution during fibrillation was kept constant below 70 °C to prevent hydrolysis of cellulose. The fibrillated cellulose was then stored in a chiller at 4 °C for further use. A solution consisting of lithium hydroxide and urea in deionized water with a ratio of 4.6:15, respectively, and precooled to −13 °C was used to dissolve the CNF. The CNF was added to the solution at a concentration of 3 to 9% and vigorously stirred at 2500 rpm for 15 min. The cooling and stirring steps were repeated several times to ensure the complete dissolution of the CNF and to achieve a constant viscosity for good shape fidelity. Before printing, the solution was centrifuged at 1500 rpm for 5 min to remove air bubbles for better extrusion.

For the carboxymethyl cellulose ink solution, a powder CMC with a ratio of 3% to 9% was added to 60 °C deionized water and stirred at 1500 rpm for 30 min until fully dissolved. The CNF solution was then added to the CMC solution, and the mixture was stirred for an additional 10 min until a constant viscosity was achieved

### 2.4. Liquid Deposition Modelling

The paste extruder was chosen for the 3D printing of cellulose nanofibrils (CNF) at different concentrations as it is well-suited for extruding the ink from the liquid phase. The Discov3ry paste extruder from Structur3d Printing, Kitchener, Canada, was used, integrated with an Ultimaker 2+ 3D printer from Ultimaker, Netherlands. The desired design was created using computer-aided design (CAD) and sliced with Ultimaker Cura 4.5 from Geldermalsen, Netherlands, following the Discov3ry configuration. A nozzle with a diameter of 0.84 mm was used for all printings at different CNF concentrations. After the design was printed, the material was regenerated using 3% H_2_SO_4_ and CaCl_2_ and then washed with distilled water before being used in protein uptake and release experiments.

### 2.5. Characterization

To gain a deeper understanding of the structure and properties of the liquid solution and 3D-printed material at various concentrations of cellulose nanofibrils, several characterizations were conducted. To analyze the morphological structure of the fractured tensile samples, Field Emission Scanning Electron Microscope (FESEM) was used (Merlin Compact, Zeiss Pvt Ltd., Oberkochen, Germany). The samples were first sputter-coated with gold before being viewed under the microscope. The functional group of the printed structure was analyzed using an attenuated total reflectance Fourier transform infrared (ATR-FTIR) (ALPHA FTIR Spectrometer, Bruker, Billerica, MA, USA) in the range of 4000 cm^−1^ to 50 cm^−1^ at a resolution of 1 cm^−1^. The crystallinity and type of cellulose fractions were examined using X-ray diffractometry (XRD) (Bruker D8 Advance, Bruker, Billerica, MA, USA) at a diffraction angle of between 5° to 60° with a scanning speed of 1°/min. The compression properties were measured using the ASTM D1621-16 standard test method for compressive properties of rigid cellular plastics, using an Instron^®^ Electro-mechanical Universal Testing Systems 3300 Series with a load cell of 100 N at a crosshead speed of 1.2 mm/min. The hydrogel was prepared with a height of 10 mm and a diameter of 24 mm.

### 2.6. Adsorption and Release of Bovine Serum Albumin

The total uptake and release of the 3D-printed CNF/CMC hydrogel was studied by immersing it in a bovine serum albumin (BSA) solution. The initial concentration of BSA varied between 10 ppm and 100 ppm, while the temperature varied between 25–60 °C and the concentration of CaCl_2_ between 2.5% to 10%. The study was conducted for 24 h without stirring, as it would have affected the shape of the 3D hydrogel. The initial and final concentrations of BSA protein were measured using a UV spectrophotometer at a wavelength of 280 nm. The final equilibrium (*q*_e_) of BSA protein uptake by the printed material was calculated using Equation (1), where *C*_0_ and *C_e_* refer to the initial and equilibrium concentrations of the BSA protein (mg/L), *V* is the volume of the BSA protein solution used (L), and *m* is the mass of the adsorbent (dried printed CNF) (g). The maximum adsorption was calculated using the Langmuir isotherm (Equation (2)).

(1) $$q_{e}=\frac{\left(C_{0}-C_{e}\right)V}{m}$$

(2) $$q_{e}=\frac{Q_{0}bC_{e}}{1+bC_{e}}$$

The release of the BSA protein from the hydrogel was conducted using phosphate buffer saline for 24 h. The percentage of uptake and release was calculated based on Equations (3) and (4), respectively.

(3) $$Uptake\;\left(\%\right)=\frac{Initial\;Concentration,\;C_{0}-Final\;Concentration,\;\;C_{F}}{Initial\;Concentration,\;C_{0}}\times 100\%$$

(4) $$Release\;\left(\%\right)=\frac{Concentration\;Release\;\left(ppm\right)}{Concentration\;Adsorbed\;\left(ppm\right)}\times 100\%$$

## 3. Results

### 3.1. Characterization

The extraction of cellulose from OPEFB fibers involves two processes, organosolv extraction for lignin removal and catalytic oxidation for further bleaching and removal. The isolation of cellulose to nanocellulose is done using mechanical disintegration techniques. The process of cellulose extraction involves firstly the organosolv extraction for the removal of lignin, followed by catalytic oxidation for further bleaching and purification. This process produces two types of products, black liquor, and cellulose pulp. The cellulose pulp undergoes a change in color from dark brown to yellowish after the catalytic oxidation process indicating successful bleaching. The weight of cellulose pulp obtained from the organosolv extraction is reduced by 39.18% from the initial weight. The weight is further reduced to 56.26% and 20.17% after catalytic oxidation I and II, respectively. In total, the amount of cellulose that can be extracted is 21.23% of the initial weight of the OEFB after both processes

**Figure 1:**
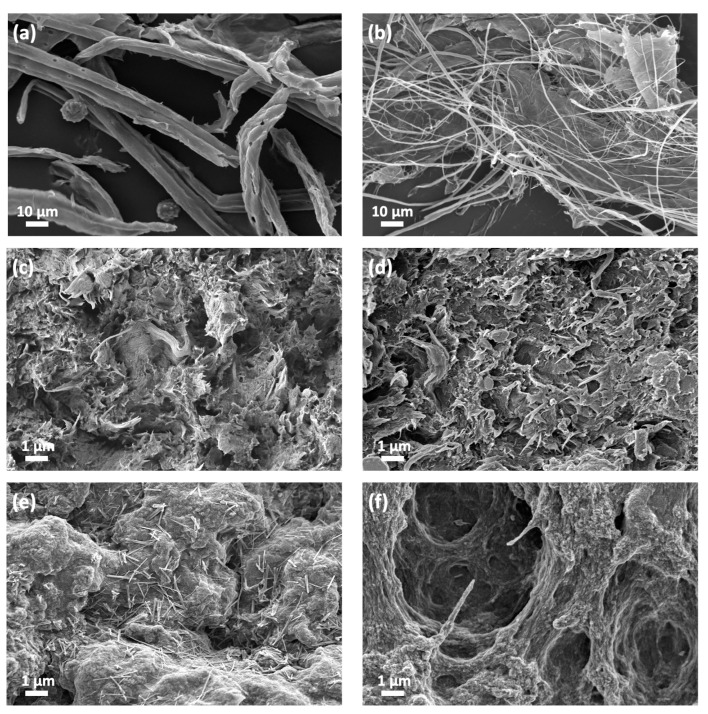
The morphological properties of the dried sample: (**a**) OPEFB fibers, (**b**) CNF, (**c**) CNF hydrogel, (**d**) Cross-sectional view of CNF hydrogel, (**e**) CNF/CMC hydrogel with crosslinker of H_2_SO_4_ and (**f**) CNF/CMC hydrogel with crosslinker of H_2_SO_4_ and CaCl_2_.

Furthermore, producing nanocellulose involves the mechanical disintegration of the cellulose pulp. Homogenizing the cellulose pulp with deionized water results in a stable and conditioned nanocellulose mixture with a concentration of 0.7 wt%. The nanocellulose produced is analyzed for its morphological properties, as seen in Figure 1a,b, confirming the type of nanocellulose produced with a diameter of ~20 nm.

To understand the properties of the hydrogel samples, several techniques were used for characterization, including morphological, chemical, and mechanical properties analysis. The morphology of each biopolymer involved in the study was analyzed using a FESEM of the dried samples. All the dried samples were tested for morphology after undergoing partial dissolution and regeneration with crosslinkers H_2_SO_4_ and CaCl_2_. The cellulose became more packed after regeneration and drying, which can lead to increased strength and flexibility of the structure.

Figure 1c,d show the surface and cross-sectional morphology of the regenerated CNF, respectively. The micrographs showed a packed regenerated CNF with fibrils and folding shapes, which can support the shape fidelity of the printed structure. Figure 1e,f show the micrographs of regenerated CNF/CMC at different crosslinkers, H_2_SO_4_ only, and both H_2_SO_4_ and CaCl_2_. The regenerated CNF/CMC using only a H_2_SO_4_ crosslinker showed a packed structure similar to the regenerated CNF. However, using both crosslinkers, a void structure could be seen compared to using H_2_SO_4_ alone. This suggests that the void structure could improve the absorptivity of the printed hydrogel, making it suitable for applications in the uptake and release of BSA solution [21]. However, it can also lead to a reduction in the strength of the material [22,23].

**Figure 2:**
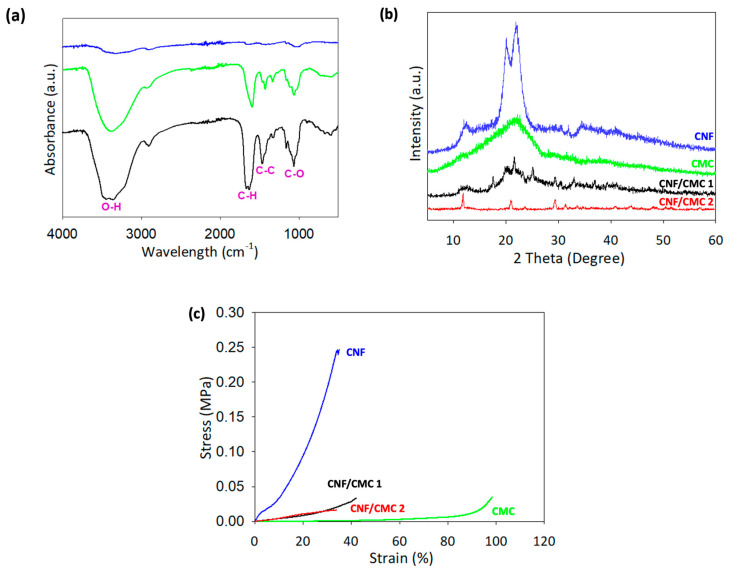
Chemical and mechanical properties of dried nanocellulose-based hydrogel of neat CNF, CMC, CNF/CMC and its crosslinkers (CNF/CMC 1: H_2_SO_4_, CNF/CMC 2: H_2_SO_4_ & CaCl_2_) for (**a**) FTIR, (**b**) XRD), and (**c**) Stress–strain curve analyses.

FTIR analysis was conducted to determine the quality and composition of the material. Figure 2a compares FTIR spectra between neat CNF, CMC, and CNF/CMC samples. The chemical analysis of the 3D-printed samples showed that broad peaks appeared at 3450 and 3350 cm^−1^ after crosslinking with H_2_SO_4_ and CaCl_2_, representing the stretching of hydrogen bonding (O-H), and 2900 cm^−1^ is also attributed to O-H stretching [24,25]. Another strong peak was observed at 1650 cm^−1,^ corresponding to the O-H bending, which was due to the interaction between cellulose and water. A peak at 1465 cm^−1^ was attributed to C-H bending scissoring motion in cellulose, 1416 cm^−1^ was attributed to O-H bending carbonyl group, and 1150 cm^−1^ corresponded to the C-C ring stretching band. A peak at 1050 cm^−1^ was attributed to the C-O stretching of alcohol from pyranose ring vibration in cellulose, and 890 cm^−1^ showed more sharp peaks due to the cellulosic β-glycosidic linkages. The composition of CNF and CMC affected peak size.

In Figure 2b, XRD analysis shows neat peaks observed at 12°, 20.3°, and 22°, while pure CMC shows a single peak at 22.6°. Both CNF/CMC with different crosslinkers showed similar peaks at 12° and 21°. Besides that, CNF/CMC 1 without CaCl_2_ crosslinking produces almost similar peak as the CMC but with more interruptions and an additional peak at 12°. The peaks observed in the XRD can be interpreted by referring to the literature [26,27,28]. The weakening cellulose peak that appeared in the neat CNF graph at 20.3° was not visible in the CNF/CMC 2 sample, but was visible in CNF/CMC 1. The disappearance of the weakening cellulose peak indicates that the CMC and CaCl_2_ improved the properties of the CNF/CMC 2 [29]. The crystallinity percentage of neat CNF was 33.5%, higher than neat CMC, which was only 20.2%. Combining CNF/CMC with CaCl_2_ in the crosslinking process produced the highest crystallinity, with 56.2% crystallinity. This suggests that the combination of CNF/CMC and CaCl_2_ crosslinking has higher strength and stiffness compared to neat CNF and CMC.

The compression test of the hydrogel revealed its ability to maintain its shape when pressure is applied. The results showed that the breaking point for each sample was similar for neat CNF and CNF/CMC with different crosslinkers, falling between 30–40% deformation of the initial height (as seen in Figure 2c). However, neat CNF hydrogel required higher stress to break, reaching 0.25 MPa at 33% deformation, comparable to previous studies on the compression of cellulose gel, which obtained 0.31 MPa at 54.75% deformation [30]. This indicates that the neat CNF hydrogel is more rigid compared to the hydrogel that includes CMC. Notably, the CMC hydrogel did not break even at 100% strain, suggesting that adding CMC improves the gel properties of the biomaterial. The Young Modulus calculated from the stress–strain curve also supports this, with CNF having the highest value at 0.0079 MPa, while the other hydrogels had lower values in the range of 0.0002–0.0009 MPa. A lower Young Modulus indicates more fracture or deformation in the material, which is evident in the CNF/CMC hydrogel [31]. To sum up, the CNF hydrogel has better strength but is lower in gel properties.

### 3.2. Nanocellulose-Based 3D Printing Parameter

The ability of nanocellulose-based biomaterial to be 3D-printed and maintain its shape was analyzed by manipulating the concentration of nanocellulose in partial dissolution. The potential of combining CNF and CMC in 3D printing was also studied, focusing on the fabrication of hexagonal shapes. The importance of crosslinking in maintaining the printed structure and ensuring full curing of the hydrogel was highlighted. The presence and different concentrations of crosslinkers significantly affected the 3D-printed hydrogel.

**Figure 3:**
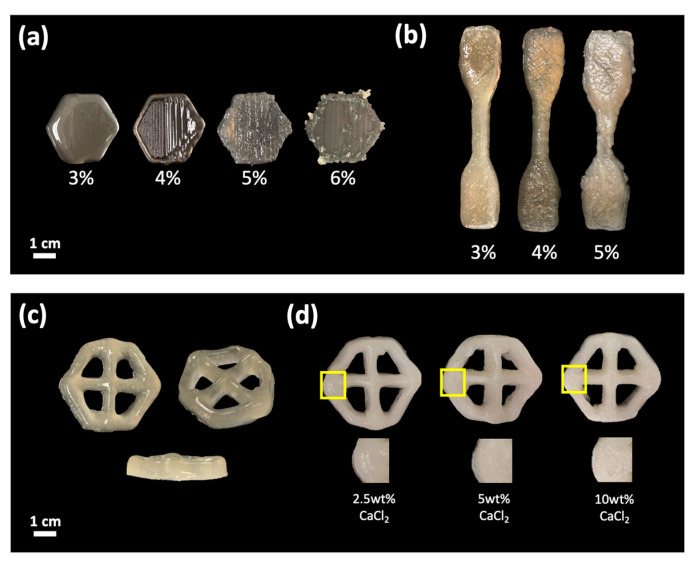
The comparison of 3D printing after partial dissolution at different concentrations of CNF: (**a**) One-layer structure design, (**b**) ASTM type IV, (**c**) Hexagonal shape with an infill of 10%, and (**d**) Different concentration CaCl_2_ as the additional crosslinker.

As shown in Figure 3a, the printing of 3% CNF could not retain the infill shapes due to the low viscosity of the ink. However, for 4% CNF, good printing shapes were produced, with clear appearances of the outer layer and infill. On the other hand, using 5% CNF resulted in a liquid ink that was too thick with a high viscosity. This led to non-smooth extrusion and a bumpy structure, caused by the higher amount of CNF compared to the LiOH/Urea solution during the partial dissolution process. The precooled solution could not fully dissolve the CNF polymer, as it was too saturated with CNF. However, a lower concentration of CNF improved the dissolution rate, and an optimal amount of CNF is required for suitability in 3D printing applications.

The designed model of ASTM Type IV (Figure 3b) shows good printing results with 4% CNF, as the shape and layer structure could be retained during the printing process. In contrast, using 5% CNF resulted in a collapse of the bottom layer and an inability to retain the shape of the outer layer. Ink viscosity is crucial in fabricating a good structure using 3D printing. Very high ink viscosity tends to cause coagulation before and during extrusion, leading to instability in layered printing and non-smooth extrusion. Therefore, an optimized viscosity is required to print layered structures with good shape fidelity.

The design model specified for the study of protein uptake and release is a hexagonal shape (Figure 3c). These hexagonal shapes happened to be more decorative than the other basic shapes. Mathematically, it has 6 sides that can minimize the perimeter and is mechanically more stable due to a higher surface tension generated from each side. The liquid printing ink uses a combination of CNF/CMC at a ratio of 62.5:37.5 with a concentration of 4.5 wt%. A combination of CNF/CMC is required as CNF promotes shape fidelity and retains shape during and after printing. Meanwhile, CMC induces gel properties due to high water content and is a thickening agent for easier printing [32,33]. The model consists of 6 layers in height with 10% of the grid infill in size 32 × 28 mm. Figure 3c shows that the combination of 4.5 wt% of CNF/CMC can print a structure with good shape fidelity. The bottom layer of the design does not collapse during the process showing a good viscosity of the ink being used. The printed sample will undergo the same coagulation process by immersion in the crosslinker solution.

The 3D-printed samples were crosslinked by immersing them in an H_2_SO_4_ and CaCl_2_ solution. Figure 3d shows a comparison of the physical appearance of the hydrogel after immersion in 2.5, 5, and 10 wt.% of CaCl_2_. However, the immersion in 5 and 10 wt.% of CaCl_2_ shows the formation of white spots, whereas, at 2.5 wt.% of CaCl_2_, the appearance of the hydrogel is similar to after crosslinking in H_2_SO_4_. The white spots may appear due to the presence of calcium and chloride ions in the crosslinkers, and the amount of white spots increases as the concentration of these ions increases.

### 3.3. Swelling and Water Absorptivity of Hydrogel

The hydrogel’s physical appearance is largely influenced by the concentration of CNF and CMC in the ink solution. The ink was also characterized by its ability to absorb water and respond to the crosslinking agents H_2_SO_4_ and CaCl_2_. As the ratio of CMC is reduced in the ink, the hydrogel’s rigidity after crosslinking increases. The hydrogel at different ratios of CNF to CMC, where it was observed that a higher ratio of CNF (87.5:12.5) resulted in better gelation compared to a ratio of 50:50. This is because a higher composition of CNF in the ink solution improves the shape fidelity of the structure due to the orientation of the crystal structure [34]. Alkaline pretreatment and partial dissolution alter the amorphous and crystalline cellulose I structure to cellulose II allomorph, thereby improving the strength and flexibility of the hydrogel [35,36,37].

Creating a hydrogel can be improved by adjusting the pH to lower the surface charge. However, using CNF alone can be problematic as they have a weak macroscopic structure, making it difficult to use them in fabricating hydrogels and biomedical applications [34]. To overcome this, adding CMC is helpful, as it is water-soluble and has anionic carboxylic groups. This improves the structure of the hydrogel and increases its absorbency, but CMC alone can result in a hydrogel with low mechanical strength [37,38,39]. A combination of CNF and CMC is necessary to achieve a hydrogel with both good mechanical strength and absorbency [39].

Using LiOH/urea to partially dissolve CNF allows for self-forming gelation through changes in temperature, pH, and time, but additional crosslinking is necessary for better observation and gelation [40]. In this study, ionic crosslinking using H_2_SO_4_ and CaCl_2_ was performed after the printing process. A low concentration of H_2_SO_4_ partially induces gelation of the printed structure, particularly of the CNF polymer chain. CaCl_2_ was used for further gelation as it is the most suitable crosslinker for CMC. The ionic crosslinking occurred through the formation of linkages between the SO^4−^ and Ca^2+^ ions and the cellulose polymer, increasing the electrostatic attraction between the anionic charge of the polymer and the multivalent cation from the crosslinker [41,42]. The resulting hydrogel structure is more stable and irreversible after cross-linkages between polymers.

The swelling capability of hydrogel and dried hydrogel was analyzed by immersing the samples in deionized water. The study was conducted for 24 h, and readings were taken at various intervals and summarized as the swelling ratio shown in Table 1. The dimensions of the hydrogel do not change significantly after immersion, but the hydrogel becomes more rigid compared to before being immersed in water. This suggests that the outer wall of the hydrogel holds water inside it and prevents an increase in dimension. As immersion time increases, there may be a break in the outer layer due to the high water content inside the hydrogel. From the tabulated data, there were only slight differences in size between the presence of H_2_SO_4_ only and both H_2_SO_4_ and CaCl_2_. As a result, the presence of H_2_SO_4_ and CaCl_2_ as crosslinkers does not significantly affect the swelling ratio of the hydrogel and dried samples.

### 3.4. Capability Uptake and Release of Protein

**Figure 4:**
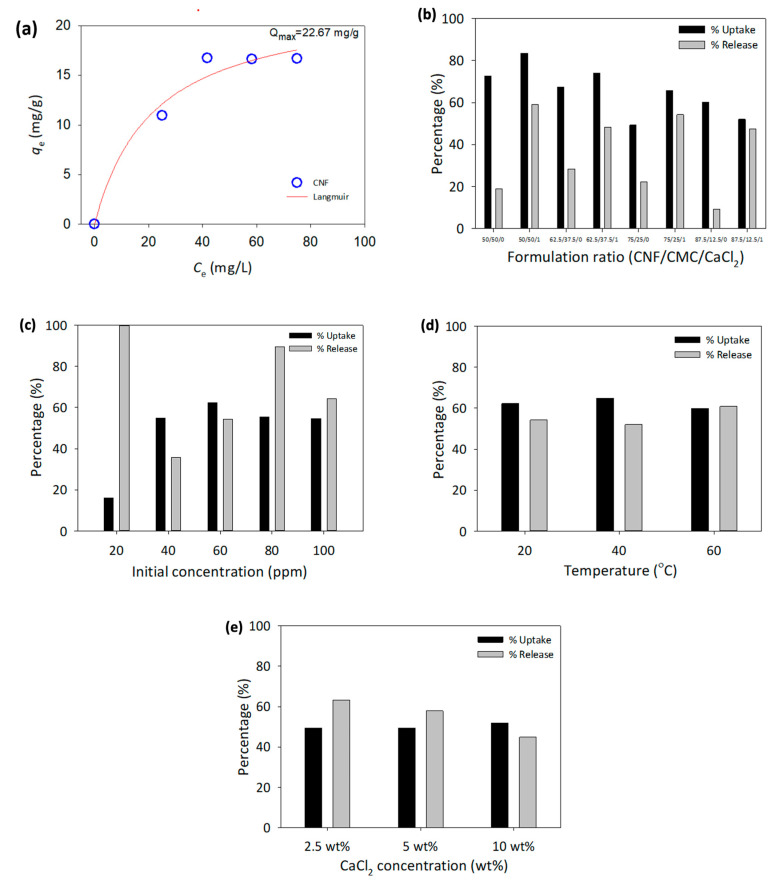
(**a**) Adsorption of BSA by neat cellulose using Langmuir isotherm model and CNF/CMC performances on uptake and release of BSA at difference (**b**) Formulation ratio, (**c**) Initial concentration of BSA, (**d**) Temperature and (**e**) Concentration CaCl_2_.

Uptake and release of protein solution by cellulose and 3D-printed hydrogel are conducted through immersing hydrogel in BSA and PBS solutions. The parameters manipulated and observed in this study were the effect of the CNF and CMC ratio, the initial concentration of BSA, temperature, and the presence of a crosslinker. The preliminary work of this study utilized cellulose pulp to explore its potential for protein uptake by immersing it in different concentrations of BSA, ranging from 60 ppm to 120 ppm for 24 h. The maximum adsorption capacity was determined using the Langmuir isotherm model, illustrated in Figure 4a, and shows that the model is appropriate for the data collected. This suggests that the adsorption of BSA by cellulose pulp is a monolayer on the surface of the structure. The total adsorption of BSA by cellulose pulp was 22.67 mg/g.

To identify the optimal ratio of CNF/CMC for protein uptake and release, various ratios of CNF and CMC were tested in the presence and absence of CaCl_2_. The initial concentration of BSA was held constant at 60 ppm at room temperature. The results, including the amount of adsorbed and released BSA, as well as the percentage of adsorption and release, are shown in Table 2. The concentration of BSA adsorption and release was determined using calibration curves of BSA and PBS solutions, as illustrated in Figure 4a. The highest adsorption percentage was 83.55% at a CNF/CMC ratio of 50:50 with the presence of CaCl_2_. On the other hand, the highest release percentage was 75.11% at a ratio of 87.5:12.5 without the presence of CaCl_2_.

As shown in Figure 4b, the presence of CaCl_2_ as a crosslinker for CMC resulted in higher protein uptake and release than using only H_2_SO_4_. This indicates that proper crosslinking for both CNF and CMC in the 3D-printed structure is crucial for the protein uptake and release process. Based on this information, an optimized ratio of CNF to CMC was chosen for 3D printing and further parameter changes. Taking factors into account, such as the viscosity of the biomaterial ink, the structure of the hydrogel, and the protein uptake and release process, a ratio of 62.5:37.5 CNF to CMC was selected for the biomaterial ink formulation, along with the use of both crosslinkers.

The 3D printing of a hexagonal model was performed using biomaterial ink with a ratio of 62.5:37.5 to investigate the impact of BSA protein solution concentration on the protein uptake and release process. The BSA concentration was varied from 20 to 100 ppm, and the experiment was conducted at room temperature using H_2_SO_4_ and CaCl_2_ as crosslinkers. The results of the protein uptake and release are presented in Figure 4c. The highest adsorption percentage was observed at 60 ppm of the initial BSA concentration at 62.30%. The highest release percentage was at 20 ppm with 100% release. The protein uptake and release by the hydrogel were greatly affected by the BSA concentration in the solution.

An investigation of the effect of temperature on protein uptake and release was conducted using a 3D-printed hydrogel with a constant ratio of CNF/CMC and the presence of both crosslinkers. The BSA concentration used was 60 ppm, based on the previous section results, as this concentration demonstrated the greatest difference in protein uptake and release. The experiments were performed at 20–60 °C for 24 h without stirring. The temperature range chosen is the most critical temperature for the protein study, where protein can be fully functionalized and has low chances for protein degradation [43]. The results in Figure 4d show that the temperature slightly impacted the protein uptake by the hydrogel. However, the release of BSA in PBS solution increased at a higher temperature. This indicates that temperature affects the process, as it increases the movement of ions and accelerates the reaction [44].

As for the effect of different concentrations of CaCl_2_, the results indicated there was no significant effect of CaCl_2_ concentration on protein adsorption, as the readings were almost similar between different concentrations (Figure 4e). However, for the release of BSA in PBS solution, the percentage of release decreased as the CaCl_2_ concentration increased.

## 4. Conclusions

The use of cellulose has a positive impact on the development and innovation of biopolymers and their biomedical applications. In this study, combining CNF and CMC improved the material properties by utilizing advanced additive manufacturing technology such as 3D printing. Complex designs with high suitability for protein uptake and release could be achieved by using this method. The presence of CMC in the ink formulation improved the protein uptake as it has a high water absorption capability. On the other hand, CNF provided shape fidelity, ensuring the maintenance of the 3D shape during and after the 3D printing process. The focus of this research was to investigate the capability of nanocellulose-based hydrogel for protein uptake and release. Additionally, CaCl_2,_ as an additional crosslinker, was necessary during curing to improve the protein uptake and release. However, concentration did not play an important role in the process. It is important to note that high temperatures during protein uptake and release directly affected the hydrogel structure. The next focus of this research field is to thoroughly examine cell viability and proliferation in nanocellulose-based hydrogel. To achieve this, researchers will conduct in vitro studies on living cells using the MTT (3-(4,5-dimethylthiazolyl-2)-2,5-diphenyltetrazolium bromide) assay, which is known for its high sensitivity and efficiency.

## Figures and Tables

**Table 1:** The percentage of the swelling ratio of CNF/CMC hydrogel and dried hydrogel after 24 h immersion in deionized water.

CNF:CMC:CaCl_2_	Swelling Ratio (%)	
	Wet	Dry
50:50:0	1.99	13.33
50:50:1	2.08	16.00
62.5:37.5:0	2.72	21.43
62.5:37.5:1	7.14	30.00
75:25:0	2.21	16.25
75:25:1	2.78	11.11
87.5:12.5:0	2.70	31.25
87.5:12.5:1	5.88	33.33

**Table 2:** The uptake and release of BSA at different ratios of CNF to CMC.

CNF:CMC:CaCl_2_	Uptake (%)	Release (%)
50:50:0	72.53	18.90
50:50:1	83.55	59.06
62.5:37.5:0	67.43	28.39
62.5:37.5:1	73.96	48.19
75:25:0	49.29	22.26
75:25:1	65.74	54.22
87.5:12.5:0	32.84	75.11
87.5:12.5:1	52.02	47.42
